# Tuberculose claviculaire révélée par une lacune osseuse

**DOI:** 10.11604/pamj.2016.24.100.9190

**Published:** 2016-05-31

**Authors:** Nadia Ben Abdelhafidh, Rym Abid

**Affiliations:** 1 Service de Médecine Interne, Hôpital Militaire de Tunis, Tunisie

**Keywords:** Os, clavicule, tuberculose, Bone, clavicle, tuberculosis

## Image en médecine

Il s'agit Mme S.S âgée de 30 ans et sans antécédents pathologiques particuliers. L'anamnèse a retrouvé la notion de consommation occasionnelle de produits laitiers non pasteurisés. Elle a consulté suite à la découverte fortuite d'une tuméfaction en regard de la clavicule droite, sans signes généraux associés. A l'examen, il s'agit d'une formation de 4 cm de grand axe, de consistance dure, indolore, sans signes inflammatoires en regard et fixe par rapport au plan profond. Le reste de l'examen somatique était normal. La biologie était sans anomalies. L'IDR à la tuberculine était phlycténulaire. La radiographie standard de l’épaule a montré une lacune osseuse du bord distal de la clavicule droite. L'IRM a objectivé une ostéolyse cortico-médullaire antéro-supérieure de la clavicule droite avec un aspect irrégulier, érodé et aminci de la corticale adjacente, épargnant l'articulation acromio-claviculaire ainsi qu'une inflammation des parties molles péri-osseuses prédominante en antérieur. La biopsie osseuse a montré de multiples lésions folliculaires faites de cellules épithélioïdes et de cellules géantes entourant de larges plages de nécrose caséeuses. Le scanner thoraco-abdomno-pelvien et la scintigraphie osseuses n'ont pas trouvé d'autres atteintes. Le diagnostic d'une tuberculose claviculaire isolée était alors retenu. La patiente était mise sous traitement antituberculeux quadruple (izoniazide, rifampicine, éthambutol et pyrazinamide) pendant deux mois puis une bithérapie (izoniazide et rifampicine) pour une durée totale de traitement de 9 mois. L’évolution clinique et radiologique était favorable. Le recul est de 18 mois.

**Figure 1:**
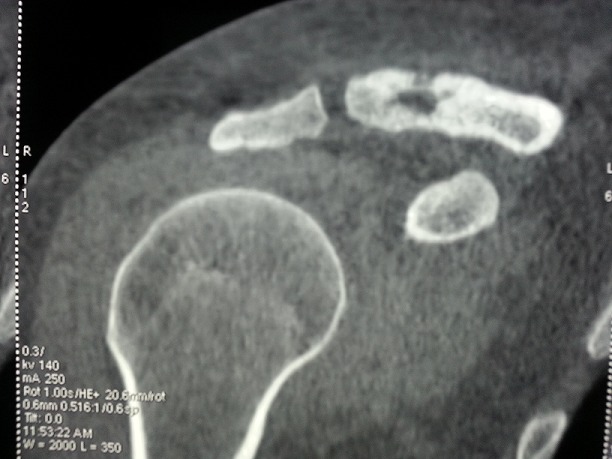
Lacune osseuse du bord interne de la clavicule

